# Collagen XV Promotes ER Stress-Induced Inflammation through Activating Integrin β1/FAK Signaling Pathway and M1 Macrophage Polarization in Adipose Tissue

**DOI:** 10.3390/ijms22189997

**Published:** 2021-09-16

**Authors:** Changxing Li, Yuexia Liu, Yizhou Li, Ruiqing Tai, Zhuwen Sun, Qiong Wu, Yongnian Liu, Chao Sun

**Affiliations:** 1 Key Laboratory of Animal Genetics, Breeding and Reproduction of Shaanxi Province, College of Animal Science and Technology, Northwest A&F University, Xianyang 712100, China; lcx1535@163.com (C.L.); nwafuliuyuexia@126.com (Y.L.); liyizhou@nwafu.edu.cn (Y.L.); tai1573861498@163.com (R.T.); szw13043277456@163.com (Z.S.); 13997126828@163.com (Q.W.); 2 Medical College of Qinghai University, Xining 810000, China

**Keywords:** FAK, Col XV, ERS, inflammation, adipose tissue

## Abstract

Collagen XV (Col XV), a basement membrane (BM) component, is highly expressed in adipose tissue, and studies have found that Col XV is related to extracellular matrix (ECM) remodeling involving in adipose tissue fibrosis and inflammation. Furthermore, the ECM is essential for maintaining normal development and tissue function. In this study, we found that Col XV is related to the endoplasmic reticulum stress (ERS) and inflammation of adipose tissue. Moreover, we found that overexpression of Col XV in mice could cause macrophages to infiltrate white adipose tissue (iWAT). At the same time, the expression of the ERS sensor IRE1α (Inositol-Requiring Enzyme-1α) was significantly up-regulated, which intensified the inflammation of adipose tissue and the polarization of M1 macrophages after the overexpression of Col XV in mice. In addition, after overexpression of Col XV, the intracellular Ca^2+^ concentration was significantly increased. Using focal adhesion kinase (FAK) inhibitor PF573228, we found that PF-573228 inhibited the phosphorylation of FAK and reversed the upward trend of Col XV-induced protein expression levels of IRE1α, C/EBP-homologous protein (CHOP), and 78 kDa glucose-regulated protein (GRP78). After treatment with IRE1α inhibitor STF-083010, the results showed that the expression of adipocyte inflammation-related genes interleukin 6 (*IL-6*) and tumor necrosis factor α (*TNFα*) significantly were decreased. Our results demonstrate that Col XV induces ER-stress in adipocytes by activating the Integrinβ1/FAK pathway and disrupting the intracellular Ca^2+^ balance. At the same time, Col XV regulates the inflammation induced by ER stress in adipocytes by promoting IRE1α/XBP1 (X-Box binding protein 1) signaling. Our study provides new ideas for solving the problems of adipose tissue metabolism disorders caused by abnormal accumulation of ECM.

## 1. Introduction 

More and more evidence suggests that metabolism-associated diseases triggered by obesity are closely associated with adipose tissue (AT) remodeling [1]. Aberrant AT remodeling can also lead to disorders of adipose tissue secretions, such as cytokines, hormones and metabolites, which can disrupt tissue metabolism and lead to imbalances in cellular metabolism [2,3,4]. Studies have found that these changes cause changes in adipose tissue function and are related to extracellular matrix (ECM) remodeling [5,6], and abnormal changes in ECM components will affect the activation of immune cells and the spread of inflammation [7]. Interestingly, the transcriptome analysis of adipose tissue in obese people found that the genes of ECM components are significantly up-regulated, and this change is closely related to the occurrence of inflammation [8]. In addition, due to the molecular structure of Col XV non-fibrous collagen, it provides a unique prelude to exploring its special properties in the ECM [9]. In animal models, it has been determined that Col XV plays an important role in many cellular processes, such as endothelial cell migration, angiogenesis and the tumor growth [10,11,12]. Osteocalcin and Col XV are both up-regulated at mRNA and protein levels by extracellular Ca^2+^, which has also previously been found in rat osteoblasts [13,14]. Studies have shown that Col XV is more highly expressed than other ECM components in adipose tissue, but there is little information about its function in adipose tissue as a histogenesis-correlated gene [12,15]. Col XV is an important component that connects the basement membrane (BM) and extramembrane matrix of adipocytes, and its expression is significantly up-regulated in differentiated 3T3-L1 cells [12,15,16]. However, the presence and function of Col XV located in BM zones in adipose tissue have not been fully recognized. This unique ECM molecule provides an essential microenvironment for adipocytes, not only affecting the biological activity of adipocytes themselves, regulating the physiological and biochemical processes within cells, but also participating in the interactions of other cells. Therefore, it is necessary to consider the role of Col XV for the treatment of obesity-related diseases caused by abnormal accumulation of ECM. 

Although these morphological and functional changes of adipocytes are related to surface receptor molecules, such as integrins, the precise molecular mechanism behind adipocyte inflammation has not been fully understood in obesity. FAK is the major downstream regulator of integrin β1, which is activated by phosphorylation after integrin β1 binds to ECM proteins, especially collagen [17,18,19]. These findings strongly indicate FAK/integrin signaling is also a key to understanding the molecular mechanisms of Col XV in adipose tissue inflammation. 

The endoplasmic reticulum (ER) is the main site for protein folding and modification in cells, and it also provides an important platform for lipid transport and metabolism. In addition, the ER is considered to be a container for intracellular calcium storage, when cells are under stress, such as in the case of hypermetabolism, ER is also indispensable in the perception of cellular stress [20,21]. In an environment of stress excess, the loss of the ER’s adaptive capacity leads to stimulation of the unfolded protein response (UPR), which includes the initiation of three transmembrane receptors, recombinant activating transcription factor 6 (*ATF6*), protein kinase R-like ER kinase (PERK), and IRE1α [22,23,24]. Studies have shown that activation of the IRE1α can recruit TNF Receptor Associated Factor 2 (*TRAF2*) onto the ER to regulate the inflammatory signals through the nuclear factor kappa-B (*NF-ΚB*) pathway [25,26,27]. Moreover, the up-regulation of intracellular Ca^2+^ concentration is involved in the regulation of downstream inflammatory responses by regulating the activation of NLR family pyrin domain containing 3 (*NLRP3*) [28]. Under the inflammatory environment, adipose tissue is infiltrated by immune cells, including macrophages, neutrophils, and lymphocytes. There are research findings that most of the macrophage population in adipose tissue of obese subjects consists of proinflammatory M1 macrophages [29].

In this report, we explore the effects of the ECM component Col XV on ERS as well as inflammation on the adipose tissue. We found that Col XV activated the FAK phosphorylation by combining with cell-membrane receptor integrin β1. In addition, the overexpression of Col XV in adipocytes disturbed the calcium balance in ER by up-regulating InsP3R (*IP3R1*); induction of ERS and ERS transducers in adipocytes led to production of inflammatory factors. Furthermore, we found that Col XV promoted M1 macrophage polarization through secretion of Interferon beta (*IFNβ*) in adipose tissue.

## 2. Results

### 2.1. Col XV Aggravates Adipose Tissue Endoplasmic Reticulum Stress 

**Figure 1:**
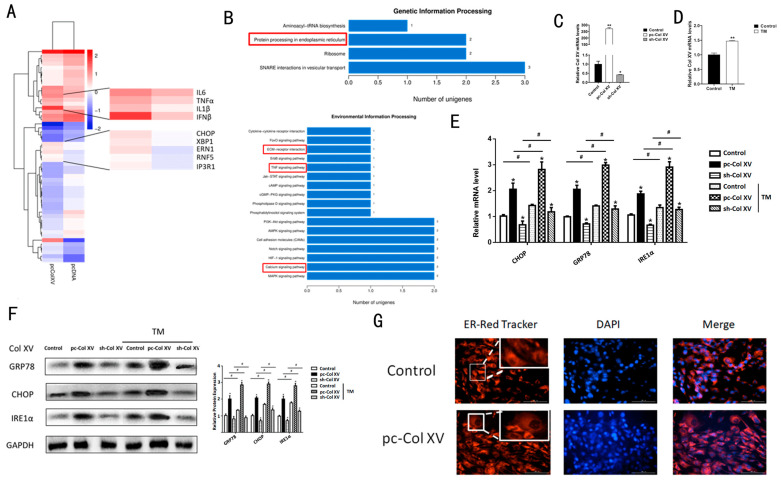
Overexpression of Col XV promotes ERS in mouse adipocytes. (**A**) Heat map of genes up- or down-regulated in response to forced expression of Col XV in mouse adipocytes, and the most severely affected genes (*n* = 4). (**B**) GO analysis of the altered genes in (**A**) (*n* = 4) (left). Enrichment analysis of altered pathway in response to forced expression of Col XV (*n* = 4) (right). (**C**) Relative Col XV mRNA levels after knockdown and overexpression of Col XV. (**D**) Relative mRNA levels of Col XV in adipocytes pretreated with or without TM (1 μM) (*n* = 4). (**E**,**F**) Relative mRNA and protein expression levels of ERS marker genes in different groups in adipocyte pretreated with or without TM (1 μM) (*n* = 4). (**G**) The morphological changes of ER detected by ER red tracker (*n* = 4). Scale bar, 100 μm. Values are means ± SEM. ^#^ or * *p* < 0.05 or ** *p* < 0.01 compared with the control group.

To investigate the effects of Col XV on adipocytes, RNA-Seq was performed to compare the transcriptome differences between the Col XV overexpression group and the control group in adipocytes. Overexpression of Col XV showed the signature genes changes of inflammation factor and ERS relates genes when significant differences of gene expression were diagrammatically displayed as a Heatmap (Figure 1A). Further Gene Ontology (GO) analysis indicated that differences in gene expression were abundant in those genes related to TNF signaling and Ca^2+^ signaling (Figure 1B). Then, we tested Col XV interference and overexpression efficiency in adipocytes (Figure 1C). Next, after TM treatment, Col XV was found to be significantly up-regulated (Figure 1D). At the same time, it was found that the overexpression of Col XV in 3T3-L1 adipocyte increases the mRNA levels involved in ERS, such as *CHOP*, *IRE1α*, *GRP78* (Figure 1E), and the protein expression level also increased (Figure 1F). To further investigate whether Col XV aggravated the adipocyte ERS, we performed the ERS model, and treated 3T3-L1 cells with tunicamycin (TM). We found there was a significant up-regulation in expression of ERS indicators in the Col XV overexpression group, whether treated with TM or not. In addition, these symbol factors exhibited an increase in the Col XV overexpression group compared to the control group after TM treatment (Figure 1E,F). ERS is often accompanied by the expansion of the ER and other changes in the shape of the ER. Here, we quantified the changes in ER signal intensity through the use of ER-tracker Red, and observed these changes with a life fluorescence microscope (Figure 1G). Observation revealed a higher fluorescence intensity of the ER-tracker and dilation of the cystic cavity with ER in the cells with overexpression of Col XV (pc-Col XV). These findings indicate that Col XV participates in and aggravates the ERS of adipocytes.

### 2.2. Col XV Induces Adipose Tissue Inflammation by Enhancing Adipocyte ERS

**Figure 2:**
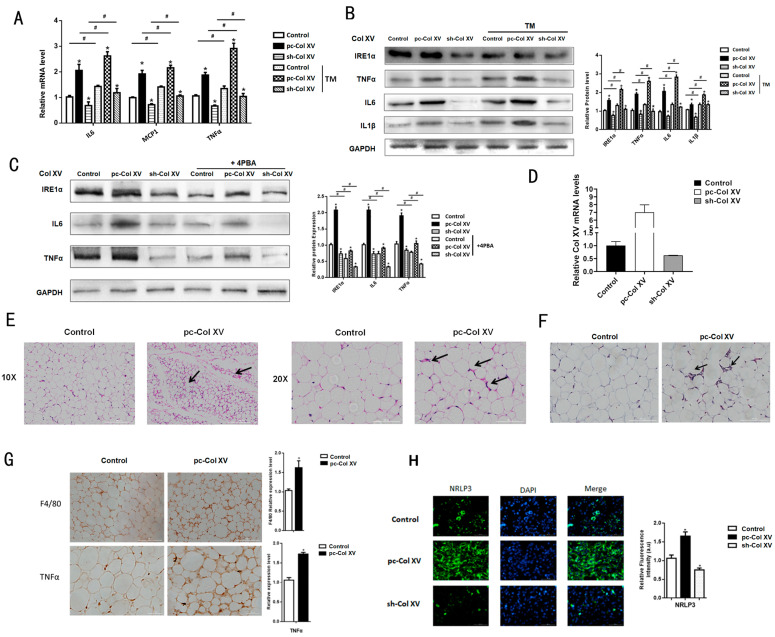
Col XV promotes adipose inflammation. (**A**) Relative mRNA and protein expression levels of inflammation marker genes *IL6*, *MCP1*, *TNFα* in mice adipocytes in control group, pc-Col XV group and sh-Col XV group with or without TM (1μM) (*n* = 4). (**B**) Relative protein expression levels of IRE1α, TNFα, IL6, IL1β in different groups in adipocytes pretreated with or without TM (*n* = 4). (**C**) Relative protein expression levels of IRE1α, TNFα, IL6 in different groups in adipocytes treated with or without 4PBA (2ug/mL) (*n* = 4). (**D**) Relative Col XV mRNA level in iWAT of treatment group mice (*n* = 4). (**E**) Images of hematoxylin and eosin staining of white adipose tissue in different groups (*n* = 4). Scale bar, 100 μm. (**F**) Representative images of Masson’s trichrome staining in iWAT Arrowheads indicate collagen fibers (*n* = 4). Scale bar, 100 μm. (**G**) Images of immunohistochemical staining for F4/80 and TNFα of adipose tissue in different groups (*n* = 4). Scale bar, 200 μm. (**H**) Fluorescence staining of NLRP3 in adipocytes in different groups (*n* = 4). Scale bar, 100 μm. Values are means ± SEM. ^#^ or * *p* < 0.05 compared with the control group.

Following RNA-Seq (Figure 1A,B), we observed that there is significant up-regulation of inflammation genes *IL6*, *IL1β* and *TNFα*. To characterize the role of Col XV in adipocyte inflammation, we transfected Col XV overexpression vector (pc-Col XV) or empty vector (pc-DNA3.1) into 3T3-L1 cells and treated them or not with TM. We observed a significant up-regulation of mRNA expression in inflammation-related genes, such as *IL6*, *MCP*1 and *TNFα*; however, TM treatment promoted a more pronounced expression of inflammatory genes (Figure 2A). Consistently, protein levels of IRE1α, TNFα, IL6 and IL1β were elevated in the overexpression group (Figure 2B). As a result of the striking character of ERS in the production of inflammation factors, especially in adipose tissue, we pretreated the cells with 4-PBA, an ERS inhibitor, and observed that 4-PBA suppresses IRE1α, TNFα and IL6 protein levels and inhibits ERS (Figure 2C). Meanwhile, we tested the interference and overexpression efficiency of Col XV in iWAT (Figure 2D). To elucidate the effects of Col XV on the niche of adipocytes, we injected mice with Ad-pc Col XV and Ad-pcDNA3.1 vector adenovirus, collected white adipose tissue, and performed HE staining. The results showed that the crown-like structure (CLS) in adipose tissue significant increases (Figure 2E). The results of iWAT Masson trichrome staining in mice treated with pc-Col XV showed that collagen deposition increased significantly (Figure 2F). The staining of histological sections of F4/80 and TNFα showed that the content of adipose tissue macrophage (ATM) in iWAT was higher due to the overexpression of Col XV group (Figure 2G). Consistent with this, the effects of Col XV on adipocyte inflammation were confirmed by the induction of NLRP3, examined by means of fluorescence microscopy (Figure 2H). These data indicate that Col XV is involved in adipose tissue inflammation through ERS induction.

### 2.3. Interaction between Col XV and Integrin β1 Is Necessary for Activation of FAK

**Figure 3:**
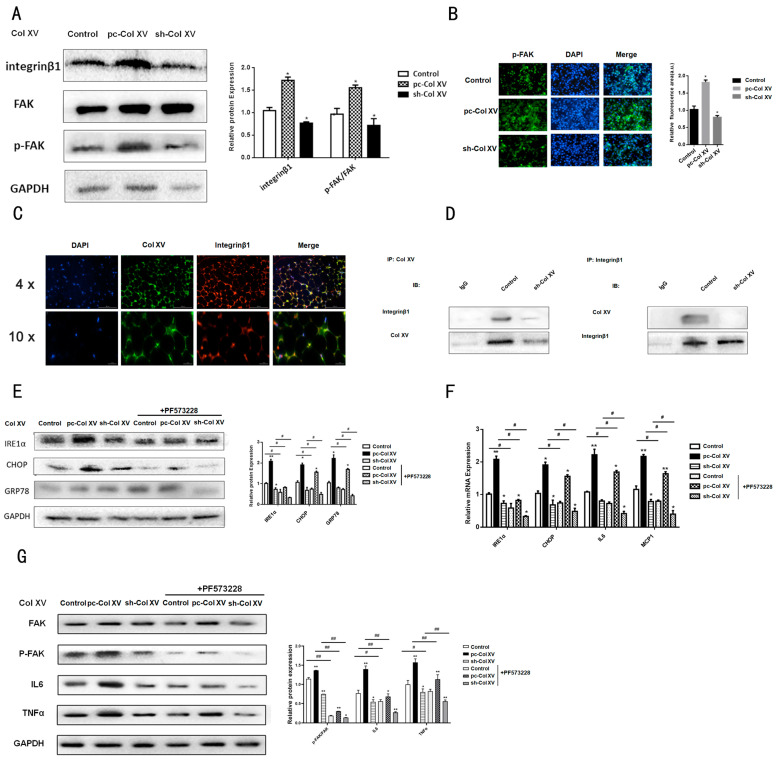
Col XV interacting with integrin β1 activates FAK. (**A**) The protein expression of integrin β1, FAK and p-FAK in different groups detected by Western blotting (*n* = 4). (**B**) Active FAK (FITC, green) in the cells is evaluated by immunofluorescence assay in different groups (*n* = 4). Scale bar, 200 μm. (**C**) Immunostaining of endogenous Col XV and endogenous integrin β1 in cells is performed for Col XV using an FITC-labeled secondary antibody, or integrin β1 using a Cy3-labeled secondary antibody (*n* = 4). (**D**) Confirmation of the interaction between Col XV and integrin β1 by co-IP (*n* = 4). (**E**,**F**) Relative protein and mRNA expressions of CHOP, IRE1α, GRP78 in different group (*n* = 4) after treatment with inhibitor of FAK PF573228. (**G**) Relative protein expression levels of p-FAK, FAK, IL6, TNFα (normalized to GAPDH) in each group (*n* = 4). Values are means ± SEM. ^#^ or * *p* < 0.05 and ^##^ or ** *p* < 0.01 compared with the control group.

FAK participates in the interaction with ECM, as a major component of focal adhesions, and activation of FAK signaling is an integrin β1 (Itg β1)-dependent effect. A recent study demonstrated that Col XV increased the phosphorylation level of FAK [30]; however, the mechanisms by which Col XV up-regulates the level of FAK phosphorylation in adipocytes has not yet been reported. As an important cell surface receptor of ECM molecules, integrin is the most likely to engage in widespread cell processes. In particular, FAK has been shown to be an important factor in delivering ECM molecules to cells through the integrin β1 receptor [31]. Therefore, it is logical to hypothesize that FAK can be a downstream pathway of Col XV-mediated ERS in 3T3-L1 cells. Next, we also probed the function of the integrin β1/FAK cascade in the IP3R1 calcium channel. Western blotting results showed that overexpression of Col XV increased the protein level of integrin β1 and promoted the phosphorylation of FAKY397 (Figure 3A). Immunofluorescence staining demonstrated the same result (Figure 3B). To further evaluate whether Col XV interacts directly with integrin β1, Col XV and integrin β1 antibodies were respectively used, and Co-IP analysis was performed in 3T3-L1 cells. As presented in Figure 3D, integrin β1 is immunoprecipitated, and integrin β1 can be pulled down by Col XV antibodies. However, the interaction of Col XV with integrin β1 is diminished by sh-Col XV treatment. Then, the immunofluorescence experiment was performed in adipocytes, and the results confirm the co-localization of Col XV and integrin β1 (Figure 3C). Meanwhile, it was also found that the protein and mRNA levels of the ERS indicators IRE1α, GRP78 and CHOP were down-regulated along with the use of FAK inhibitors (PF-573228) (Figure 3E,F). In addition, the expression of inflammation-related factors (IL6, MCP1, TNFα) are inhibited by treatment with PF-573228 (Figure 3G). Thus, integrin β1/FAK signaling may serve as the main pathway for regulation of Col XV to adipocyte ERS and inflammation.

### 2.4. Col XV Triggers Adipocyte ERS by Disrupting Intracellular Ca^2+^ Homeostasis through IP3R1

**Figure 4:**
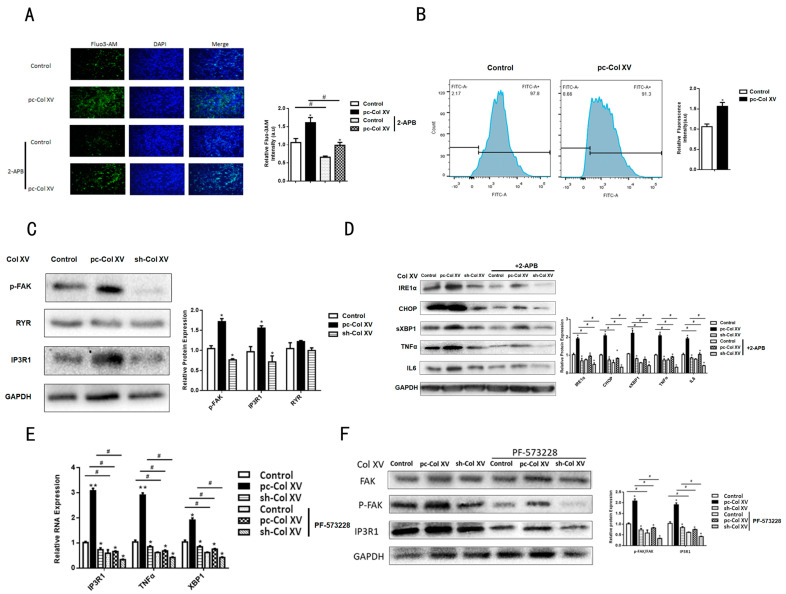
Col XV disrupts intracellular Ca^2+^ homeostasis through IP3R1. (**A**) Analysis of [Ca^2+^] based on Fluo-3 AM mean fluorescence intensity after treatment assessed by fluorescence microscopy (*n* = 4). Scale bar, 200 μm. (**B**) FCM analysis of Cytosolic Ca^2+^. (**C**) Relative protein expressions of p-FAK, RYR, IP3R1 in adipocytes in the control group, pc-Col XV group and sh-Col XV groups (*n* = 4). (**D**) Relative protein expressions of IRE1α, CHOP, sXBP1, TNFα, IL6 in different groups incubated with 2APB (*n* = 4). (**E**) Relative mRNA levels of *IP3R1*, *TNFα*, *XBP1* in adipocytes treated with PF-573228 (*n* = 4). (**F**) Relative protein levels of FAK, p-FAK and IP3R1 in adipocytes treated with or without PF-573228 (*n* = 4). Values are means ± SEM. ^#^ or * *p* < 0.05 and ** *p* < 0.01 compared with the control group.

We further investigated potential mechanisms that could explain the influence of Col XV on the ERS of adipocytes. The previous RNA-sequence revealed a significant change of *IP3R1* mRNA expression with the overexpression of the Col XV group compared to the control group; the GO analysis also exhibited a distinct difference in the genes that converged on related molecules involved in Ca^2+^ signaling pathway (Figure 1A,B). We speculate whether the imbalance of ER Ca^2+^ homeostasis plays a critical role in ERS caused by Col XV. The concentration of Ca^2+^ in the cytoplasm was detected using a Fluo-3AM, and we observed a significant increase in the concentration of Ca^2+^ in the cytoplasm in the pc-Col XV-treated group (Figure 4A). Similar results were obtained on the basis of flow cytometry measurements (FCM) (Figure 4B). Next, we studied the ER-related proteins and Ca^2+^ channels in the ER membrane during the calcium release process induced by Col XV-1. At the same time, we used the Ca^2 +^ -free culture medium containing 50 μM EGTA to explore the possibility of increased Ca^2+^ concentration in the cytoplasm rooted in extracellular medium. However, the concentration of Ca^2+^ did not significantly change in the cytoplasm. This indicates a primary role for Ca^2+^ release from the intracellular Ca^2+^ stores, but not through extracellular Ca^2+^ influx. As shown by our results, compared with the control group, pc-Col XV treatment was able to increase the mRNA and protein expression levels of IP3R1, but not the expression levels of RYR (Figure 4C,E). To determine whether the increase in Ca^2+^ in pc-Col XV-treated cells was caused by ER Ca^2 +^ -related channels, we adopted a special calcium channel antagonist, 2-aminoethoxydiphenyl borate (2-APB), to inhibit InsP3R. Indeed, as shown in Figure 4A, 2-APB treatment lessened the augmentation of Ca^2+^ concentration caused by Col XV. Additionally, Western blotting indicated that 2-APB treatment decreased the expression levels of the ERS-related factors CHOP, IRE1α and XBP1 (Figure 4D). However, the administration of inhibitors of FAK interdicted the up-regulation of IP3R1 caused by Col XV overexpression (Figure 4E,F). These results suggest that *IP3R1* had a direct impact on the loss of ER Ca^2+^ homeostasis induced by Col XV.

### 2.5. IRE1α/XBP1 Branch Pathway of ERS is Predominantly Activated during Col XV-Induced Adipocyte Inflammation

**Figure 5:**
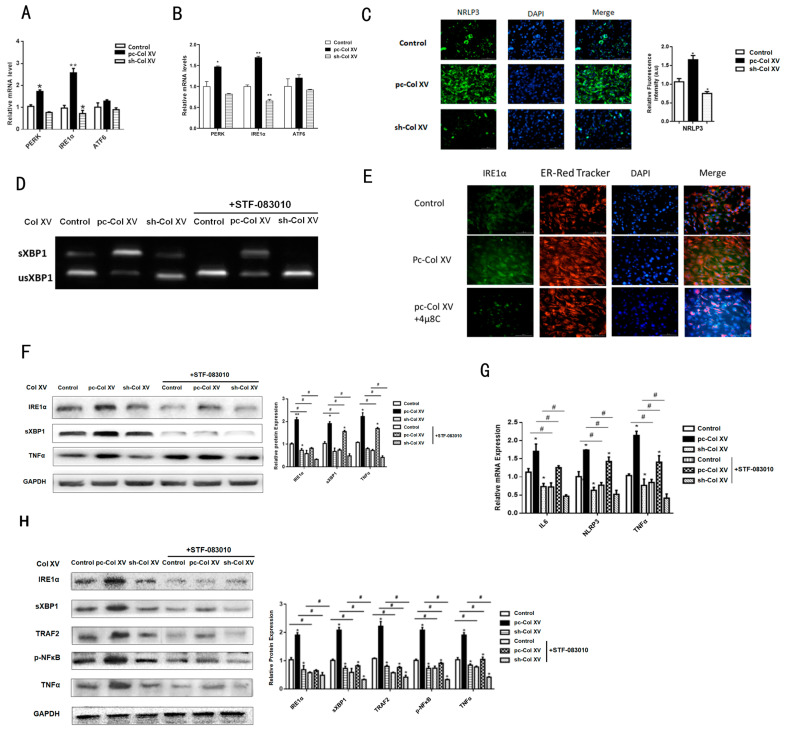
IRE1α/XBP1 branch pathway participates in Col XV-induced inflammation. (**A**) Relative mRNA expression levels of *PERK*, *ATF6* and *IRE1α* in different groups on adipocytes (*n* = 4). (**B**) Relative mRNA expression levels of *PERK*, *ATF6* and *IRE1α* in different groups on iWAT (*n* = 4). (**C**) Representative images of IRE1α immunofluorescent staining in adipocytes (*n* = 4). Scale bar, 200 μm. (**D**) XBP1 mRNA splicing assay (RT-PCR) of indicated cells treated with PCR fragments corresponding to the uXBP1 or sXBP1 forms (*n* = 4). (**E**) Representative images of ER-tracker staining in different groups with or without inhibitor of IRE1α 4μ8c (Red) in adipocytes (*n* = 4). Scale bar, 200 μm. (**F**) Relative protein expression levels of IRE1α, sXBP1 and TNFα in different groups on adipocytes (*n* = 4). (**G**) Relative mRNA expression levels of *IL6*, *TNFα*, *NLRP3* in adipocytes in different groups with or without STF083010 (*n* = 4). (**H**) Relative protein expression levels of IRE1α, sXBP1, TNFα, TRF2, p-NF-κB in adipocytes in different groups with or without inhibitor of IRE1α STF-083010 (*n* = 4). Values are means ± SEM. ^#^ or * *p* < 0.05 and ** *p* < 0.01 compared with the control group.

The breakdown of calcium balance in the ER brings about the abnormal increase of unfolded proteins and the occurrence of ERS. Therefore, our next objective was to identify the UPR signaling pathways that could be involved in the inflammation gene up-regulation caused by Col XV. We further characterized the molecular branch signals with respect to ERS engaged in inflammation by detecting the genetic changes of the major membrane baroreceptor molecule in three pathways of UPR. Compared with the control group, *IRE1α* has a greater increase in expression levels than *ATF6* and *PERK* in the Col XV overexpression group (Figure 5A). The expression levels of *IRE1α*, *ATF6* and *PERK* in the iWAT of the mice in the treatment group were consistent with the results in the cells (Figure 5B). Multiple studies have shown that the IRE1/XBP1 pathway plays a key role in the process of pro-inflammatory cytokine production stemming from ERS [32,33]. To gain insight into whether the IRE1α-XBP1 pathway is regulated by Col XV in adipocytes, we observed an increase of IRE1α detected by immunofluorescence in the Col XV overexpression group (Figure 5C), which is consistent with previous results showing an increase in transcription levels (Figure 1A). Strikingly, IRE1α inhibitors (STF-083010) completely block the splicing of XBP1 (a target of IRE1α) in adipocytes (Figure 5D), and the inhibition of IRE1α by treatment with 4μ8c led to significant remission of ERS (Figure 5E). Consistent with these results, Western blotting results showed significant up-regulation of IRE1α and sXBP1 after treatment with pc-Col XV compared to control (Figure 5F). We next observed that IRE1α inhibition blunted the mRNA expression of inflammatory markers *IL6*, *TNFα* and *NLRP3* driven by Col XV (Figure 5G). Furthermore, the production process of the inflammatory factor was coupled to an increase in IRE1α, which was also accompanied by slight TNFα activation of the NF-κB cascade (Figure 5H). IRE1α has been identified to be capable of recruiting TRAF2 into ER under stress, and further coupling with IKKβ to form a complex that activates the NF-κB pathway during ERS [34,35,36]. These results indicate that the IRE1α/XBP1 branch of ERS is related to Col XV-induced adipose tissue inflammation.

### 2.6. Col XV Aggravates Adipose Tissue Inflammation Depending on M1 Macrophage Polarization

**Figure 6:**
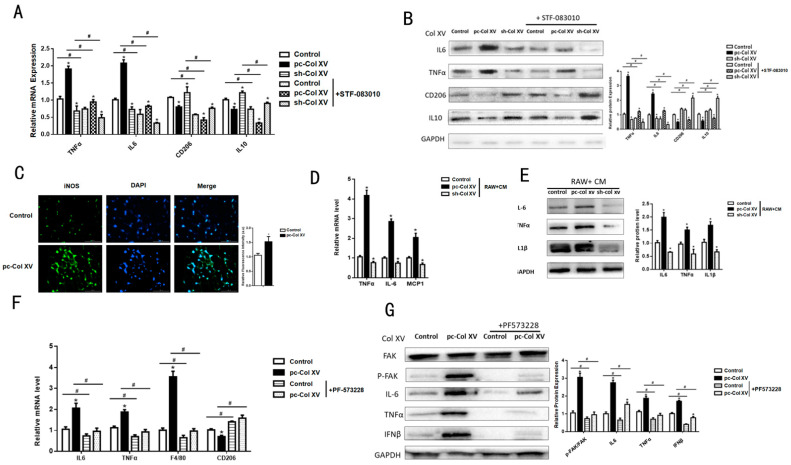
Col XV promotes adipose tissue M1 macrophage polarization. (**A**,**B**) 3T3-L1 cells are co-cultured with Raw264.7 cells by a trans-well (cell contact independent, soluble mediator driven) system. Relative mRNA and protein expression levels of IL6, TNFα, CD206, IL10 in RAW246.7 cells in different groups with or without the inhibitor of IRE1α STF083010 (*n* = 4). (**C**) Representative immunofluorescence method for iNOS in different groups (*n* = 4). Scale bar, 200 μm. (**D**,**E**) Relative mRNA expression levels of TNFα, IL6, MCP1 and protein expression levels of TNFα, IL6, IL1β in RAW246.7 cells incubated with the adipocyte control medium with or without the inhibitor of IRE1α STF-083010 (*n* = 4). (**F**,**G**) Relative mRNA expression levels of TNFα, IL6,F4/80,CD206 and relative protein expression levels of FAK, p-FAK, IL6, TNFα, IFNβ in mice iWAT in different groups (*n* = 4). Values are means ± SEM. ^#^ or * *p* < 0.05 compared with the control group.

We found that adipose tissue inflammation was accompanied by infiltration of macrophages in previous results. To investigate the mechanism of ATMs in response to Col XV-induced adipose tissue inflammation, we examined the interconnectedness among the macrophages and adipocytes. The adipocytes and Raw264.7 cells were indirectly co-cultured in a trans-well system. Prior to the co-culture, 3T3-L1 cells were treated with empty vector, pc-Col XV, or sh-Col XV. We observed an increase in the protein and mRNA levels of M1 macrophage genes IL6 and TNFα in the group treated with the overexpression Col XV, and, instead of suppression of CD206, IL10 expression (Figure 6A,B). In addition, the iNOS expression of Raw264.7 macrophage incubated with conditioned medium (CM) treated with pc-Col XV was elevated by means of immunofluorescence (Figure 6C). Moreover, we used adipocyte-CM treated with empty vector, pc-Col XV, and sh-Col XV to incubate the Raw264.7 macrophage. The qRT-PCR and Western blotting results of IL6 and TNFα were in line with previous results (Figure 6D,E). Interestingly, the M1 macrophage polarization markers were inhibited by STF-083010 (Figure 6A,B). To discuss whether FAK activation participates in M1 macrophage polarization, we injected the inhibitor of FAK PF-573228 into the mouse’s inguinal region and isolated iWAT. As a result, we found it obstructed the affection of Col XV on macrophage polarization (Figure 6F,G). Therefore, it is more likely that Col XV promotes adipose tissue inflammation through alternative activation of macrophage polarization.

### 2.7. IFNβ Secretion from Adipose Tissue Induced by ERS Plays a Role in M1 Macrophage Polarization

**Figure 7:**
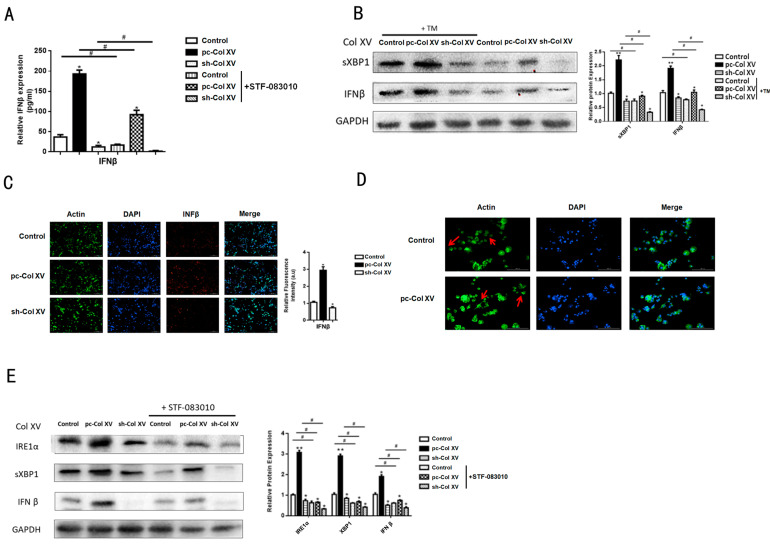
Col XV increases ERS-mediated IFNβ secretin. (**A**) *IFNβ* expression level in medium supernatant in different groups with or without STF-083010 detected by ELISA (*n* = 4). (**B**) Relative protein expression levels of sXBP1, IFNβ in adipocytes in different groups with or without TM (*n* = 4). (**C**) Images of adipocytes INFβ stain by immunofluorescent staining in different groups (*n* = 4) Scale bar, 200 μm. (**D**) Representative images showing morphology of cells after labeling for F-actin (phalloidin-FITC, green) and nuclei (DAPI; blue) (*n* = 4). Scale bar, 100μm. (**E**) Relative protein expression levels of IRE1α, sXBP1, IFNβ in adipocytes in different groups with or without STF-083010 (*n* = 4). Values are means ± SEM. ^#^ or * *p* < 0.05 and ** *p* < 0.01 compared with the control group.

On the basis of RNA-Seq results showing that overexpression Col XV in adipocytes promotes *IFNβ* production, *IFNβ* is known to be involved in ERS [37,38,39]. We next executed an expression measurement experiment to affirm its impact in inflammation response. We collected the supernatant of adipocytes after transfection with pc-Col XV, then observed the increase of *IFNβ* expression detected by ELISA (enzyme-linked immuno sorbent assay) (Figure 7A). A previous study reported that intracellular stress can magnify *IFNβ* induction in response to UPR signaling [40]. To determine whether ERS induction also induces IFNβ expression in vivo, we treated 3T3-L1 adipocytes with TM. Compared with the control group, TM induced an increase of sXBP1 and IFNβ protein levels (Figure 7B). The immunofluorescence results showed an increase of IFNβ in iWAT (Figure 7C). RAW246.7 cells incubated with CM displayed a change in morphology (Figure 7D). It has been reported that NF-κB, (ATF2)/c-Jun and IRF3 may participate in regulating the transcription of IFNβ [41,42,43]. When inhibiting IRE1α (STF-083010), the IFNβ protein level was decreased (Figure 7E). This result is in line with a previous study, in which ERS enhanced the activation of the signaling pathways controlling IFNβ, and XBP1 contributed to ERS-amplified IFNβ production [37]. Taken together, Col XV promotes M1 macrophage polarization through the secretion of *INFβ* by adipose tissue.

## 3. Research Highlights

- Col XV aggravates adipose tissue ERS;
- Interaction between Col XV and integrin β1 is necessary for activation of FAK;
- Col XV triggers adipocyte ERS by disrupting intracellular Ca^2+^ homeostasis through IP3R1;
- IFNβ secretion from adipose tissue induced by ERS plays a role in M1 macrophage polarization;
- Col XV promotes ERS induced adipose inflammation through FAK/integrin β1 signaling pathway and M1 macrophage polarization in adipose tissue.

## 4. Discussion

Adipose tissue inflammation induced by obesity is often accompanied by abnormal accumulation of ECM or the process of ECM remodeling. In our previous study, we identified that the expression level of Col XV in adipose tissue and differentiated adipocytes was significantly up-regulated in HFD-induced mice [12]. Diet-induced obesity is strongly associated with inflammation and immune response [29]. In this study, we demonstrated that the overexpression of Col XV promotes adipocyte inflammatory factor expression and inflammation in iWAT of mice via morphological observation. Our study suggests that accumulation of Col XV as the important component of ECM might play a pivotal role in obesity-induced inflammation beginning in adipose tissue.

The proper biological activity of the ER is essential in maintaining the metabolic homeostasis, function and survival of cells [12]. So far, ER has been regarded as a key regulator of systemic metabolic balance. Excessive nutrition, inflammation, and other metabolic diseases all result in the normal function of the ER being impaired, as well as the activation of three classical pathways of the UPR response [44,45]. The role of ER in the regulation of metabolism in hepatocytes and β cells has been well studied, but knowledge of the molecular mechanisms in adipocytes are still inadequate. In this study, we assessed the most important role of Col XV in inflammatory genes in adipocytes at the transcriptional level and found the alterations in the genes of ERS signaling. We found that IRE1α was significantly increased by Col XV. Furthermore, the ER morphology changed in adipocytes treated with the overexpression of Col XV. Obesity is often accompanied by the failure of the UPR response to maintain the function of the ER, especially in tissues with active metabolism, such as the liver, leading to the occurrence of ERS and inflammatory reactions, and eventually leading to the destruction of the metabolic stability of the system [46]. Then, we performed a TM-induced ERS model and ERS blocker 4-PBA in adipocytes to verify that the molecular processes of inflammation induced by Col XV was associated with ERS. Here, we found TM-induced ERS and inflammatory factor production in adipocytes, which is in line with previous reports [47]. Therefore, we determined that Col XV might promote adipose tissue inflammation through the production of inflammatory factors induced by adipocyte ERS.

Here, we found that Col XV promoted adipocyte ERS along with the activation of Integrin β1/FAK signaling. Previous reports have indicated that FAK is a fatal link in the cascade of integrin signals, which regulates growth and survival of tumors [48]. FAK is most recognized for its central role in integrin signaling, which responds to stimuli from the ECM. Moreover, we demonstrated that Col XV increased TM-induced inflammation in white adipose tissue. Therefore, this work builds on previous studies showing the importance of the ECM in obesity and diabetes. The deficiency of key ECM components such as collagen Vα3, matrix metalloproteinase14 or tissue inhibitor of matrix metalloproteinase2 can reduce adiposity and impair glucose homeostasis in vivo [49]. Further validation of Col XV within intracellular metabolic homeostasis is warranted. A previous study indicated that the FAK-PI3K-calcium (Ca^2+^) signaling pathway was activated via coupling to α3β1 integrin [50]. We demonstrated that Col XV could combine with integrin β1 to active FAK, sh-Col XV and FAK inhibitor PF573228, apparently diminishing the phosphorylation of FAKY397. Here, we detected the increase of Ca2+ channel IP3R1 expression in adipocytes after the overexpression of Col XV. Consistent with previous studies, the activation of FAK was able to interact with IP3R1 at adjacent ERS induced by Ca^2+^ signaling [51].

It has been found that IRE1 is involved in the regulation of chronic inflammation in ATMs, providing a reliable clue for making further improvement in our understanding of inflammatory diseases regulated by the ERS signaling pathway [52]. Here, we identified that IRE1α/XBP1 signaling plays a crucial part in Col XV-induced adipocyte inflammation caused by ERS. We found higher levels of IRE1α expression than PERK or ATF6 in association with adipocyte ERS, which was appreciably correlated with increased FAK activation. In addition, as an important junction between adipocytes and macrophages, there is a particular structure in obese adipose tissue in association with inflammation called CLS [53]. We observed that Col XV led to the increase of CLS in iWAT. ATMs play an important role in administrating metabolic inflammation, and ERS contributes to macrophage activation [54]. We found that Col XV promoted M1 macrophage marker expression along with the infiltration of macrophages in white adipose tissue in mice. This observation is supported by the phenomenon of CLS surrounding dead adipocytes, which exhibits a pronounced pro-inflammatory classic activation of the M1 phenotype [55,56].

In summary, we discovered that the structural element Col XV in adipocyte BM is critical for the maintenance of the metabolism of the steady state of adipocytes. Meanwhile, excessive Col XV activates the integrin β1/FAK axis, inducing abnormal Ca^2+^ accumulation in adipocyte cytoplasm, ultimately leading to sustained adipose tissue inflammation. Moreover, the overexpression of Col XV in adipose tissue promotes the polarization of M1 macrophages. Therefore, our results describe a new relationship among Col XV, ERS and inflammation. Our work could clear the path forward for regulatory mechanisms of adipose tissue ECM component expansion and adipose tissue inflammation, with the aim of seeking novel approaches for therapies for obesity-related disease. 

## 5. Materials and Methods

### 5.1. Experimental Animals

C57BL/6J male mice were purchased from the Fourth Military Medical University (Xi’an, China). In addition, we raised and handled experimental animals in strict accordance with relevant regulations and animal ethics regulations. Recombinant Col XV (pc-Col XV) adenovirus overexpression vector or interference vector (sh-Col XV) was subcutaneously injected into mice every 2 days for a total of 2 weeks. Mouse iWAT and BAT were collected to study the physiological changes of tissues. For the study of inflammation in vivo, iWAT was administered to STF-083010 (1 mg/kg, Selleck, Houston, TX, USA) or PF-573228 (1 mg/kg, Selleck, Houston, TX, USA) in 0.9% saline (MACKLIN, Shanghai, China) for 7 days, then it was sampled and studied further.

### 5.2. Isolation and Culture of Adipocytes

The 3T3-L1 cell line was obtained from China Shanghai Baili Company (Shanghai Bioleaf Biotech Co., Shanghai, China), and the Raw264.7 cell line was obtained from China Wuhan Procell Biotech Co. Primary adipocytes were separated and cultivated as previously described [57]. The pre-adipocytes were treated with overexpression Col XV adenovirus vector (pc-Col XV), adenovirus interference vector of Col XV (sh-Col XV) (24 h or 48 h at the titer of 1 × 10^9^ IFU/mL); the process of adipocyte differentiation is described in the previous study.

3T3-L1 and Raw264.7 cell lines were cultivated in DMEM (Gibco, Shanghai, China). Pre-adipocytes were separated from mice epididymal adipose tissue and cultivated in 1:1 F12/DMEM (Gibco, Shanghai, China). In the co-culture system, adipocytes were washed after the denotative process and seeded in the upper chamber, while activated Raw264.7 cells were cultivated in the lower chamber.

### 5.3. RNA-Seq Analysis

Experimental adipocytes were separated from the inguinal white adipose tissue (iWAT) of 6-week-old mice. The adipocytes were infected with purified overexpressing Col XV adenoviral vector (pAd-Col XV) or a blank adenoviral vector (control). The total RNA from the adipocytes was treated with various adenovirus vectors acquired by RNAiso Reagent (Takara, Beijing, China, D312), and the RNA-seq assay was performed as previously described [58]. RNA sequencing was carried out using a Hiseq 4000 instrument (Illumina, San Diego, CA, USA). Real-time analysis was used for base calling.

### 5.4. Drug Treatments

Adipocytes were incubated with 4-phenylbutyrate (4-PBA, 50 nM, Sigma-Aldrich, MO, USA, SM0309) for 12 h. TM (1 µM, MACKLIN, Shanghai, China) was used to construct an ERS model (Sigma-Aldrich, MO, USA); STF083010 (50 nM, Selleck, Houston, TX, USA) and PF573228 (1 μM, Selleck, Houston, TX, USA) were prepared to treat the cells after transfection. IP3R channel was suppressed with 2-Aminoethoxydiphenylborane (2-APB) (50 nM, Abcam, MO, USA).

### 5.5. Cultivation of Macrophage with Conditioned Media

The process for the culture of cells was performed as previously described [59]. Adipocytes were treated with pc-Col XV or sh-Col XV and the control vector for 24 or 48 h, and then the supernatant of the conditioned medium was collected and mixed with the RAW246.7 cells stimulated by LPS (10 ng/mL, Solarbio, Beijing, China) and IL4 (10 ng/mL, Acrobiosystems, Shanghai, China), respectively, for 6 h. Finally, total RNA was obtained from RAW264.7 cells and processed for qPCR quantification of pro-inflammatory or anti-inflammatory (CD206) markers.

### 5.6. Immunohistochemistry (IHC)

Adipose tissue was cut into 5 μm sections for dyeing with eosin (Solarbio, Beijing, China) and hematoxylin (Solarbio, Beijing, China) after processes of fixation, dehydration and wax immersion. Next it was washed three times with PBS, 5 minutes each time, and the sections were then blocked with 4% rabbit serum (WOLSEN, Shenzhen, China) for 1 hour. Next it was washed three times with PBS, 5 min each time, and incubated with the antibody for 1.5 h at room temperature and then with the corresponding secondary antibody for 1 hour. It was then washed with PBST three times, 5 minutes each time, and observed through a comparison microscope. 

### 5.7. Immunofluorescence

Adipose tissue sections or cells for staining were fixed with 4% paraformaldehyde (MACKLIN, Shanghai, China) and permeated with 0.1% Triton x-100 (Solarbio, Beijing, China), and then sealed with 5% BSA (WOLSEN, Shenzhen, China) for 45 minutes. Slices or cells were incubated with primary antibody against NLRP3 (Abcam, Wales,, England), IL1β (Abcam, Wales, England), IRE1α (Abcam, Wales, England), IFNβ (Abcam, Wales, England) and p-FAK (Abcam, Wales, England) at 37 °C for 2 hours. After three 30-min washes with PBS, samples were stained for 4 h at room temperature with fluorescent secondary antibodies (Abcam, Shanghai, China), followed by 10 min of DAPI staining for nucleus visualization.

### 5.8. Quantitative Real-Time PCR (qRT–PCR)

Total RNA was isolated using TRIpure Reagent kit (Takara, Dalian, China) according to the manufacturer’s instructions. cDNA was generated by using the High Capacity cDNA Reverse Transcription Kit (Takara, Dalian, China). The process for qRT-PCR was performed as described previously [60].

### 5.9. Western Blot Analysis

Western blotting was carried out using an SDS-PAGE Electrophoresis System. Adherent cells or adipose tissue extracts were prepared and transferred to PVDF membranes. The primary antibodies for this experiment were as follows: anti-GAPDH (AB2000, Abways, Shanghai, China), anti-Col XV (ab202554, Abcam, Shanghai, China), anti-CHOP (YM3668, Immuno Way, Suzhou, China), anti-GRP78 (ab21685, Abcam, Shanghai, China), anti-IRE1α (ab124945, Abcam, Shanghai, China), anti-IL-6 (ab100712, Abcam, Shanghai, China), anti-MCP1 (ab100712, Abcam, Shanghai, China), anti-IL-1β (ab100712, Abcam, Shanghai, China), anti-CD206 (ab125028, Abcam, Shanghai, China), anti-CD163 (YM6146, Immuno Way, Suzhou, China), anti-TNFα (11948P, Cell signaling, Massachusetts, USA), anti-pFAK (ab81298, Abcam, Shanghai, China), anti-FAK (ab40794, Abcam, Shanghai, China), anti-Integrin β1 (ab24693, Abcam, Shanghai, China), Horseradish peroxidase anti-rabbit (Sigma-Aldrich, Shanghai, China) or anti-goat (Sigma-Aldrich, Shanghai, China) was used as secondary antibody. See Antibody Information Sheet (Appendix A) for antibody details.

### 5.10. Co-Immunoprecipitation (IP) and Immunoblotting

The cells were lysed in the lysis buffer as previously described [60]. A Co-Immunoprecipitation kit (Thermo Fisher Scientific, Shanghai, China) was used for IP analysis. IP analysis was carried out by cultivating total cell lysate (1 mg) with the demonstrative antibodies at 4 °C for 12 h. Then agarose beads were mixed with the previous complex and stored for another 4 h for incubation. Beads were washed once with the lysis buffer and three times with washing buffer, and then eluted by boiling in SDS sample buffer, followed by Western blot.

### 5.11. Enzyme-Linked Immunosorbent Assay (ELISA)

The protein levels of IFNβ in conditional media obtained from adipocytes were measured using commercial ELISA kits from Sigma (Sigma-Aldrich, St. Louis, MO, USA) in accordance with the manufacturer’s instructions.

### 5.12. Intracellular Ca^2+^ Measurement

The intracellular calcium content was measured with the fluorescent dye Fluo-3AM (Biotechnology, Nanjing, China), of which Flu-3AM is suitable for drugs under the action of intracellular esterase. Fluo-3 AM uses a concentration range of 1–5 μM depending on different cell types and densities. The stock solution was dissolved with PBS to the concentration of the working solution to be used. The cells washed with PBS were incubated with the appropriate concentration of Fluo-3 AM solution at 37 °C for 45 min, and next were washed with PBS. Then, the fluorescence intensity was observed under a fluorescence microscope or fluorescence detection was performed on a flow cytometer.

### 5.13. ER-Tracker Red Staining

(1) The ER-Tracker Red (Beyotime, Beijing, China) stock solution was diluted with PBS into a working solution in accordance with the instructions for use.
(2) Dyeing
  - ash the cells carefully with an appropriate amount of buffer such as PBS. For the staining of suspension cells, please refer to the staining method of adherent cells.
  - Mix the prepared staining solution with the cells, and incubate for 30 min in the incubator.
  - Remove the ER-Tracker Red staining working solution, and carefully wash the cells 1–2 times with PBS.
  - Observe the cells through a fluorescence microscope and analyze changes in fluorescence intensity.

### 5.14. Statistical Analysis

Statistical calculations were performed using SAS v8.0 (SAS Institute, Cary, NC). Statistical significance was determined by using the one-way ANOVA test. Comparisons among individual means were made by Fisher’s Least Significant Difference (LSD) post hoc test after ANOVA. Data are presented as mean ± SD; *p*< 0.05 is considered to be significant.

## Abbreviations

Extracellular Matrix	ECM
Collagen XV	Col XV
Endoplasmic Reticulum	ER
Endoplasmic Reticulum Stress	ERS
focal adhesion kinase	FAK
Inositol-Requiring Enzyme-1α	IRE1α
X-Box Binding Protein 1	XBP1
C/EBP-Homologous Protein	CHOP
78kDa Glucose-Regulated Protein	GRP78
Interleukin 6	IL-6
Tumor Necrosis Factor α	TNFα
Recombinant Activating Transcription Factor 6	TAF6
Protein kinase R-like ER kinase	PERK
TNF Receptor Associated Factor 2	TRAF2
Nuclear Factor kappa-B	NF-Κb
NLR Family Pyrin domain containing 3	NLRP3
Interferon beta	IFNβ
Adipose Tissue	AT
Inguinal White Adipose Tissue	iWAT
Basement Membrane	BM
Unfolded Protein Response	UPR
InsP3R	IP3R1
4-Phenylbutyrate	4-PBA
Gene Ontology	GO
Tunicamycin	TM
Crown-Like Structure	CLS
Adipose Tissue Macrophage	ATM
Integrin β1	Itg β1
2-Aminoethoxydiphenyl Borate	2-APB
Conditioned Medium	CM
Flow Cytometry	FCM

## Appendix A

**Antibody Information Sheet**		
**Antibody**	**Model**	**Company**
anti-GAPDH	AB2000	Abways
anti-Col XV	ab202554	Abcam
anti-CHOP	YM3668	Immuno Way
anti-GRP78	ab21685	Abcam
anti-IRE1α	ab124945	Abcam
anti-IL-6	ab100712	Abcam
anti-MCP1	ab100712	Abcam
anti-IL-1β	ab100712	Abcam
anti-CD206	b125028	Abcam
anti-CD163	YM6146	Immuno Way
anti-TNFα	11948P	Cell signaling
anti-pFAK	ab81298	Abcam
anti-FAK	ab40794	Abcam
anti-Integrinβ1	ab24693	Abcam
Horseradish peroxidase anti-rabbit	ATM	Sigma-Aldrich
Horseradish peroxidase anti-goat	Itg β1	Sigma-Aldrich
